# Parent-Child Attachment and Social Adaptation Behavior in Chinese College Students: The Mediating Role of School Bonding

**DOI:** 10.3389/fpsyg.2021.711669

**Published:** 2021-10-28

**Authors:** Haowen Yin, Suning Qian, Fengqiu Huang, Huibin Zeng, Casper J. P. Zhang, Wai-Kit Ming

**Affiliations:** ^1^Faculty of Psychology, Tianjin Normal University, Tianjin, China; ^2^Kangda College of Nanjing Medical University, Lian Yungang, China; ^3^School of Foreign Languages, Xuzhou University of Technology, Xuzhou, China; ^4^Department of Public Health and Preventive Medicine, School of Medicine, Jinan University, Guangzhou, China; ^5^School of Public Health, The University of Hong Kong, Hong Kong SAR, China; ^6^Department of Infectious Diseases and Public Health, Jockey Club College of Veterinary Medicine and Life Sciences, City University of Hong Kong, Hong Kong SAR, China

**Keywords:** parent-child attachment, social adaptation, school bonding, college students, intermediate role

## Abstract

Family and school are two main places for adolescents to develop socialization, which can be contributed by good parent-child attachment and school bonding. Earlier studies suggested that parent-child attachment played an important role in promoting the formation of high-level school bonding, which is also likely to influence social adaptation. This study aimed to explore the relationship between parent-child attachment and social adaptation, and the mediating role of school bonding. Using stratified cluster sampling, 1,440 college students were first randomly selected from four universities and then stratified by specialty with a balance between genders and grades. Participants voluntarily participated in this study and completed questionnaires including the Parent-Child Attachment Scale, School Bonding Scale, and Social Adaptation Scale. Finally, a total of 1,320 college students were included in the analysis (59.5% female; aged 18–24years, M_age_=20.39±1.52years). Data analysis and structural equation modeling were conducted using SPSS 22.0 and AMOS 23.0. The results indicated that the overall level of parent-child attachment in females (*M*=75.72, *SD*=12.36) was significantly higher than that of males (*M*=73.71, *SD*=12.68; *F*=8.22, *p*<0.01). Difference was also found between sibling status (*F*=13.90, *p*<0.001), and the only-child (*M*=76.16, *SD*=12.72) scored significantly higher than their counterparts (non-only children, *M*=73.60, *SD*=12.19). Parent-child attachment was positively correlated with social adaptation (*p*<0.01) and school bonding (*p*<0.01), while school bonding was also positively correlated with social adaptation score (*p*<0.01). School bonding played a partial intermediate role in the relationship between parent-child attachment and social adaptation (*β*=0.15). Our research identified a direct influence of parent-child attachment and an indirect influence *via* school bonding on social adaptation among college students.

## Introduction

Social adaptation is a process in which individuals actively regulate their behaviors to achieve a state of balance and coordination with their external environment (Kahle, 1984; Mahoney and Bergman, 2002). The criteria for evaluating an individual’s social adaptation usually include two aspects: the individual’s internal state of psychological harmony and the other is the individual’s external behavioral performance. The former is mostly measured by depression, anxiety, withdrawal, self-esteem, autonomy, psychological well-being, optimism, and responsibility, which can be collectively referred to as implicit adaptation; the latter is mostly measured by aggressive behavior, substance abuse, hyperactivity, pro-social tendency, acting efficiency, active coping, and other behaviors, which can be collectively referred to as explicit adaptation (Nie et al., 2008). Social adaptation is a multidimensional and very broad concept with developmental specificity. According to critical developmental task theory (Waters and Sroufe, 1983), as individuals age, the major domains of their social life change significantly, and their socially adaptive behaviors inevitably change; the importance and appropriateness of a particular psychological quality or behavior may vary at different times in an individual’s development. From the perspective of the “domain-functional” theoretical model, social adaptation is mainly divided into four domains: self-adaptation, interpersonal adaptation, behavioral adaptation, and environmental adaptation, which cover the main domains of college students’ development, as well as two functional states of positive and negative adaptation. A good social adaptation is a comprehensive reflection of a high level of positive adaptation and a low level of negative adaptation (Zou et al., 2012).

The level of social adaptation reflects the social and psychological maturity of an individual. College students are at an important age of psychological maturity in life, and their social adaptation status is not only related to their psychologically healthy development but also affects interpersonal harmony and social stability. Therefore, good social adaptation status is of great significance to the growth and development of college students. College students’ social adaptation is influenced by a variety of factors (Howell, 2003). Previous studies have shown that social adaptation was correlated to specific external factors such as parent-child relationship (Jin et al., 2011; Xu et al., 2018; Busygina et al., 2019) and school bonding (Maddox and Prinz, 2003; Demanet and Houtte, 2012; Yin et al., 2019). Parent-child attachment quality is an important indicator of the parent-child relationship (Bowlby, 1988), which refers to a state of strong and lasting emotional connection formed interaction between individuals and their parents. Previous studies have found that parent-child attachment was strongly associated with social adaptation (e.g., low depression and self-esteem; Belsky et al., 1984; Laible et al., 2004). Another critical factor influencing social adaptation is school bonding. School bonding, referred to a student’s “connection” to school and all aspects of academic life, has been found associated with various developmental and adaptive outcomes (Maddox and Prinz, 2003). For college students, as school represents a major life domain, school bonding is particularly critical for their adaptation (Liljeberg et al., 2011; Demanet and Houtte, 2012).

Social adaptation is a widely-known indicator of mental health, and it is vital to improving our understanding of the relationship between parent-child attachment, school bonding, and social adaptation. Several studies have reported that the parent-child attachment is directly or indirectly associated with many psychological outcomes (Eijck et al., 2012; Waite and Creswell, 2014; Ştefan and Avram, 2017). Moreover, both the developmental systems theory and the individual developmental context theory hold that the parent-child attachment is the basis for the development of school bonding. Therefore, the present study aimed at exploring the relationship between parent-child attachment and social adaptation, and the mediating role of school bonding.

### Direct Relationship Between Parent-Child Attachment and Social Adaptation

In the early stage of socialization of the child, the most important nurturing institution is his family. Later on, different educational institutions would join the family and lay the foundation for his mastery of the objective and social environment. The dominant sphere of the child’s development is the cognitive sphere of his interrelations with the subjects of his activity. That is why the process of his social adaptation should be organized through interconnection with other people and the goals of this interconnection. It should be characterized by acquiring correct actions in different situations (Busygina et al., 2019). Parents are the child’s first teachers in this process, and they are extremely important for the child’s development of social adaptation. Bioecological Systems theory suggests that over time, children’s characteristics interact with proximal processes and the environment to influence the development of their adaptive capacities (Bronfenbrenner and Ceci, 1994; Bronfenbrenner and Morris, 1998). Parent-child attachment is a very important proximal process in individual development and will continuously affect children’s psychological and behavioral development (Bradford and Lyddon, 1993).

Attachment is a universal human need that leads to the formation of intimate emotional bonds, according to Bowlby (1988). The parent-child attachment is deemed as a kind of deep, enduring, intimate, and stable emotional connection that forms in the process of interaction between individuals and their parents (Ramsdal et al., 2015), which is also known as “parental attachment.” Early parent-child attachment is an important symbol of individual emotional socialization. Whether there is an attachment between infants and their parents the quality of attachment can directly affect the formation of infants’ emotion, social behavior, personality characteristics, and basic attitude towards human communication (Engels et al., 2001). Compared with students who experience insecure attachment, students who have established secure attachment relationships with their parents exhibit higher self-esteem, better academic performance, and social skills, lower emotional problems such as depression, anxiety, and alienation, and fewer adaptation problems such as hostility, aggressive behavior, and social pressure (Engels et al., 2001; Ooi et al., 2006). Based on previous studies, it may be established that parent-child attachment is closely pertained to social adaptation.

It is obvious that good parent-child attachment can create a safe, warm, and supportive condition for individuals and continue to affect individuals to establish a trustful and harmonious relationship with others (Laible et al., 2000). Ruhl et al. (2015) study showed that attachment with parents in early life is associated with individuals’ psychosocial outcomes in adulthood. Moreover, Huff and colleagues also suggested that parent-child attachment can significantly predict children’s social adaptation (Huff, 2001), while Paredes et al. (2014) found the type of secure attachment has a positive role in promoting social adaptation. According to the theory of the internal working model of attachment, individuals’ early attachment (usually with parents) will be internalized into the psychological representation of self, others, and interpersonal relationships (Maltais et al., 2017). Once the internal working model is formed, it becomes stable and will have a far-reaching impact on individuals’ future interpersonal relationships and various psychosocial behaviors. Besides, attachment theory holds that high-quality attachment can enable individuals to interact with people beyond personal networks and promote social adaptation and personality development (Goldner and Scharf, 2011; Nie et al., 2016). Therefore, it could be concluded that parent-child attachment was directly correlated to social adaptation. Good parent-child attachment quality or secure parent-child attachment was a protective factor for social adaptation.

Moreover, the family is a context that involves “gender relations” (Hinde et al., 1988). In their review, Collins and Russell (1991) conclude that understanding mother-child and father-child relationships often “requires a distinction between the gender of the offspring and the gender of the parents.” Thus, parental and child gender contribute to the parent-child relationship (Hinde et al., 1988). We are also interested in gender differences in the attachment because gender differences in maternal and paternal attachment are very complex in studies of Western cultures (Kenny and Rice, 1995). Given the different social cultures and Chinese cultural values regarding gender roles, we are particularly interested in understanding whether parental attachment is stronger in females than in males. In addition, some previous studies have shown higher levels of parent-child attachment in females than males (O'Koon, 1997; Wilkinson, 2004; Song et al., 2009), we sought to determine whether this would be true among Chinese college students.

Previous studies have shown that the number of siblings (family size) also affects parent-child attachment (Kitamura et al., 1998). Some studies have found differences in parent-child attachment between only and non-only children. As the number of siblings increases, individuals perceive their parents to be less loving, more rejecting, and more neglectful, and perceptions of parental care may decrease (Liu, 2009; Li et al., 2020). In China, with the implementation of the three-child policy, increasing families have more children, and family relationships are being reorganized. Parents’ roles have therefore changed, and they are facing new experiences and challenges, which will have a direct impact on the environment of family education, the quality of parent-child attachment, and many other aspects. Therefore, it will be interesting to explore the differences in parent-child attachment between only-child and non-only child families (multi-child families).

### Indirect Relationship Between Parent–Child Attachment and Social Adaptation

Notably, the relationship between parent-child attachment and social adaptation may be direct or indirect (Jin et al., 2010; Kim et al., 2013; Ding et al., 2020a). In the current study, we attempted to explain the relationship between parent-child attachment and social adjustment by providing an explanatory mechanism. According to developmental situation theories, the development of individual results from the joint environmental influence from family and school (Lerner et al., 2011). In addition to the family, school is also a pivotal environment in the process of youth socialization. Therefore, we explored an important affective factor that has been repeatedly found to be associated with adaptation, scilicet, school bonding (Oelsner et al., 2011; Rovis et al., 2015; Yin et al., 2019). School bonding is defined as a positive emotional connection created by teachers and peers within the school, which is embodied by students’ sense of belonging (Maddox and Prinz, 2003). It also helps form identity in the school in terms of cognition, emotion, and behavior (Schneider et al., 2008; Liljeberg et al., 2011). It has 2-fold meanings, namely, their perceptions toward school and their perception of people’s (teachers, classmates, and other adults) attitudes toward themselves (Liljeberg et al., 2011). If people in school accept, respect, and support them, and they would experience a sense of belonging and security in the school. Strong evidence indicates that students’ relationships with schools have a powerful influence on their health behaviors. For example, students who have a feeling of intimacy with the school (i.e., a strong sense of connection with the school) will be more adapt to the life of the school (Oldfield et al., 2018). A study conducted by McNeely et al. (2002) showed that school bonding had a protective effect on violence and anti-social behaviors. Therefore, school bonding may be a prominent intervention target for school-based interventions, and it is also an important way to reduce student problem behaviors. Regardless of how school bonding is conceptualized, high levels of school bonding have been positively associated with positive student outcomes, including academic achievements, such as increased academic motivation, self-efficacy, academic performance, and positive academic mood (Wentzel, 1998; Roeser et al., 2000; Oelsner et al., 2011), and negatively associated with low levels of problem behaviors (e.g., substance use, truancy, bullying, fighting, stealing, and vandalism; McNeely et al., 2002; McNeely and Falci, 2004; Liljeberg et al., 2011). All of these factors are indirect manifestations of social adaptation in individual psychological and behavioral terms. However, the strength of the impact and contribution of school bonding to students’ nonacademic adaptation (e.g., social adaptation) is uncertain. At the same time, previous studies have been focusing more on the preventive effect of school connection on problem behaviors and less on its positive aspects, such as promoting individual social adaptation. In this study, we aimed to examine the direct relationship between school bonding and social adaptation.

On the other hand, family and school are two main places for college students to establish socialization. Previous studies showed that parent-child attachment was associated with students’ school bonding (McNeely et al., 2002; Mikulincer and Shaver, 2015). Good parent-child attachment and school bonding are the keys to ensure the healthy development of college students. Given that family environment preceding school environment in the course of development, the level of parent–child attachment in adolescents or young adults could also affect their subsequent school bonding. This notion appears to be supported by empirical evidence and theoretical assumption. Several studies have found that an adolescent’s sense of school bonding might stem from a healthy reciprocal relationship in the family context (e.g., secure parent-child attachment) during the socialization process (Shochet et al., 2007; Kocayörük and Şimşek, 2016). Attachment theory also points out that the quality of parent-child attachment will affect individual personality and the degree of socialization of interpersonal communication in school or university settings (Bretherton, 1985). Good parent-child attachment, for example, can help college students obtain positive self-perception and empathy abilities, and this will be beneficial for the establishment of positive relationships with teachers and classmates in school to obtain comfort, support, and acceptance, which can ultimately form good school bonding. These theoretical and practical evidence have already inferred that parent-child attachment is the key prerequisite and foundation for forming a good school connection.

As an important factor affecting the healthy development of students, school bonding has been frequently explored in research as a mediating factor, and several empirical studies have provided evidence for it. For example, Maddox and Prinz (2003) found that school bonding mediates the relationship between family influence and adolescent substance use, delinquency, and other problem behaviors. Another study indicates that school bonding partially mediates the relationship between race and risk behavior (Yang and Anyon, 2016). In addition, other studies have found that school bonding played a mediating role in the relationship between family connectedness and future orientations (e.g., academic achievement; Crespo et al., 2013). Based on the above studies, the relationships among parent-child attachment, school bonding, and social adaptation are complex and interrelated. In the present study, we examined whether school bonding could serve as a mechanism to elucidate the indirect relationship between parent-child attachment and social adjustment among college students.

### The Present Study

For college students, college life is in the transitional period of leaving their families and entering society. During this period, students’ relationships with parents can have an important impact on their development. Mutually positive relationships between parents and children have important consequences for how children approach and engage in the school environment (Kocayörük and Şimşek, 2016). In China, with the implementation of the three-child policy, family structure and family relationships have gradually changed. Many single-child families have become multi-child families. With the increase in the number of children, parents no longer focus on one child. The way of communication and interaction between parents and children and the parent-child attachment has changed correspondingly. Studies have found that after the second child is born, first-born children with low parent-child attachment levels will have poor adaptability due to anxiety and worry. On the contrary, first-born children with high parent-child attachment levels will trust and understand their parents more and can better adapt to the changes brought about by the birth of the second child (Li et al., 2020). Therefore, poor parent-child attachment is not conducive to effective social adaptation or psychological well-being for the individuals. In this context, it is important to investigate parent-child attachment and social adjustment among Chinese college students. Therefore, the purpose of this study was to explore the cross-sectional associations between parent-child attachment, school bonding, and social adaptation among Chinese college students. Clarifying the relationships among these variables may help implement specific prevention strategies to improve college students’ social adaptation.

**Figure 1:**
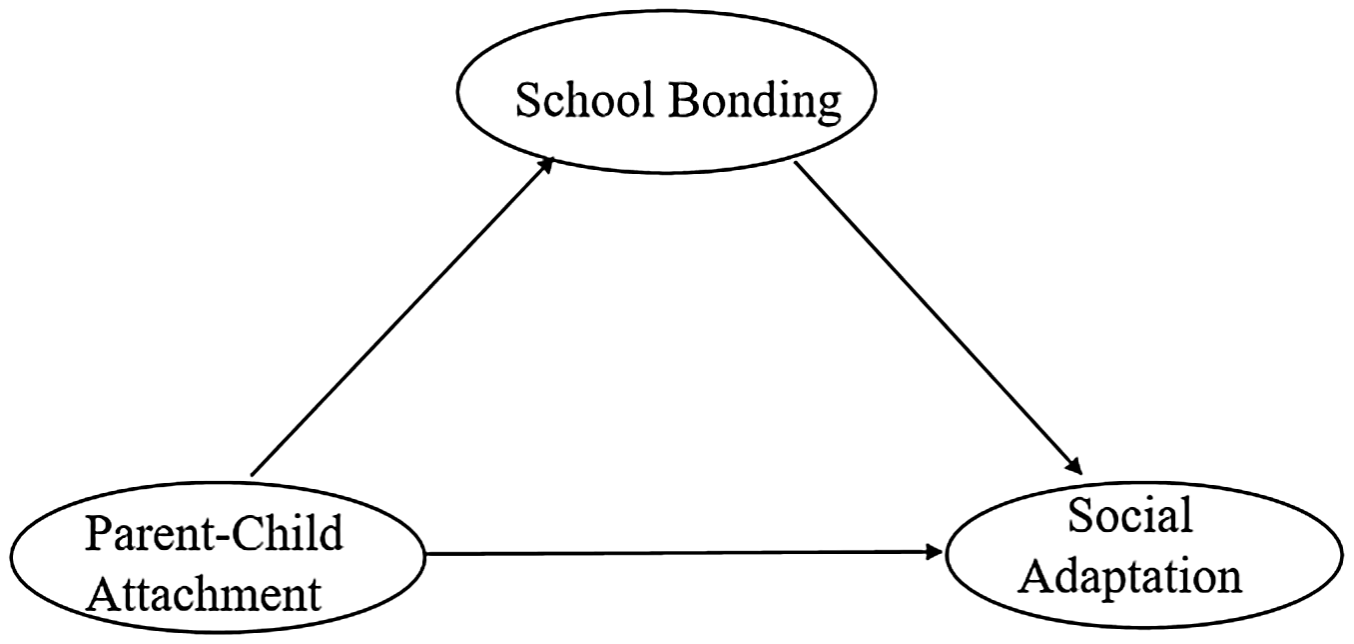
Hypothetical model.

Based on the above literature review and theories, we propose the following research hypothesis: First, there are significant differences in the parent-child attachment relationship between gender and whether or not an only child. The level of parent-child attachment of girls is higher than that of boys, and the level of parent-child attachment of the only child is higher than that of the non-only child (H1). Second, the parent-child attachment was directly correlated to social adaptation (H2). Third, the parent-child attachment was positively correlated with school bonding (H3). Fourth, school bonding was positively correlated with social adaptation (H4). Finally, parent-child attachment is indirectly related to social adaptation, and school bonding moderates the relationship between parent-child attachment and social adaptation (H5). Based on the hypotheses above, the hypothetical model of the current study is shown in Figure 1.

## Materials and Methods

### Study Design and Participants

This cross-sectional survey was conducted in May 2019. To obtain a representative sample of college students in Jiangsu province, we employed a multi-stage cluster sampling method. We first purposively selected four universities located in four cities in Jiangsu [two, respectively from southern (Suzhou and Changzhou) and northern (Xuzhou and Lianyungang) in consideration of economic difference]. Each selected university was stratified by specialties (literature and history, science and engineering, medicine, and other specialties), and one class within the specialty was subsequently sampled as a unit, proportional to the number of students in different grades (from freshman to senior) in the whole sample. An invitation letter, including the information about the study purpose and content, was initially emailed and posted to each university administrator. Upon obtainment of permission from the university administrators, trained research assistants recruited participants onsite and obtained participants’ initial consent by word of mouth. Given that the questionnaire was paper-based, participants were also required to indicate their consent for participation by ticking the box at the beginning of the questionnaire. After completing the questionnaires, each participant was thanked verbally. Data collection was administered collectively during class hours. Among 1,440 initially-contacted students, 1,320 college students consented to participation. The response rate was 91.7%. Participants’ age ranged from 18 to 24years old and the mean age was 20.39years (*SD*=1.52), of which 534 (40.5%) were male and 786 (59.5%) were female.

### Measures

Each student was invited to complete a series of questionnaires that measures parent-child attachment, school bonding, and social adaptation status.

#### General Information Questionnaire

The information is compiled by ourselves, including gender, grade, major, and sibling status (only child or not).

#### Parent-Child Attachment Scale

The level of parent-child attachment was measured using the Chinese version of the abbreviated Inventory of Parent and Peer Attachment revised by Wang and Song (2012). This scale encompassed three sub-scales of mother-child attachment, father-child attachment, and peer attachment. Each sub-scale contained 10 questions measuring three dimensions (trust, communication, and alienation). A five-point scale from 1 (never) to 5 (always) was employed. Higher scores indicated the higher quality of attachment. Since peer attachment was not involved in this study, only two subscales of mother-child attachment and father-child attachment were selected. The total scores of the two sub-scales were added to evaluate individuals’ perceptions of their relationship with their parents. Previous studies have shown that this scale also has good reliability and validity among Chinese college students (Zhang et al., 2018; Ding et al., 2020b; Wu et al., 2020). The fitting indexes of confirmatory factors analysis were based on the following metrics: (a) comparative fit index (CFI) and Tucker-Lewis index (TLI), best if greater than or equal to 0.90; and (b) root mean squared error of approximation (RMSEA), best if less than or equal to 0.08 (Hu and Bentler, 1999). The results of confirmatory factor analysis showed that the main fitting indexes of the mother-child attachment sub-scale: CFI=0.96, TLI=0.95, and RMSEA=0.07; the main fitting index of father-child attachment sub-scale: CFI=0.95, TLI=0.93, and RMSEA=0.08, which indicated that the questionnaire had high structural validity (Brosseau-Liard et al., 2012; Brosseau-Liard and Savalei, 2014). In this study, McDonald’s omega coefficients for parent-child attachment, mother-child attachment, and father-child attachment were 0.90, 0.82, and 0.86, respectively.

#### School Bonding Scale

We used the School Bonding Scale revised by Yu et al. (2011) to measure school bonding. This scale includes 10 items, measuring three aspects of student support (four items), teacher support (three items), and school belonging (three items). Sample items for each dimension are “Your classmates can share happiness and sadness with you” (student support), “I think the teachers were very caring and supportive.” (teacher support), and “In school, I feel happy and safe” (sense of school belonging). A five-point scoring is used, ranging from 1 (totally disagree) to 5 (totally agree). Higher scores indicate higher levels of school bonding. Previous studies have shown that this scale had good reliability and validity among Chinese college students (Gao et al., 2020). The results of confirmatory factor analysis showed that the main fitting indexes of school bonding scale: CFI=0.98, TLI=0.97, and RMSEA =0.08, which indicated that the questionnaire had high structural validity (Brosseau-Liard et al., 2012; Brosseau-Liard and Savalei, 2014). McDonald’s omega coefficients of the whole scale in the current study were 0.93.

#### Social Adaptation Status

A 50-item questionnaire revised by Zou et al. (2012) was used to assess social adaptation status. This scale comprised four dimensions: self-adaptation, interpersonal adaptation, behavioral adaptation, and environmental adaptation. Each dimension is divided into positive and negative aspects. A five-point scoring is adopted, ranging from 1 (not at all suitable) to 5 (perfectly suitable). This questionnaire has been proved in previous studies to be applicable to Chinese adolescents (Zou et al., 2012; Miao et al., 2018) and college students (Ma and Guo, 2017). The results of confirmatory factor analysis showed that the main fitting indexes of school adaptation scale: CFI=0.90, TLI=0.90, and RMSEA =0.07, which indicated that the questionnaire had high structural validity (Brosseau-Liard et al., 2012; Brosseau-Liard and Savalei, 2014). McDonald’s omega coefficients of the whole scale were 0.84.

### Statistical Analysis

Descriptive statistics were calculated for socio-demographic characteristics (gender, major, grade, and sibling status) of the whole sample. Two-way ANOVA and independent sample *t*-test were used to analyze the difference in parent-child attachment between gender (men vs. women) and sibling status (only-child vs. non-only child).

Correlations between parent-child attachment, school attachment, and social adaptation were also examined. Structural equation models with maximum likelihood estimation were then used to examine the mediating role of school bonding between parent-child attachment and social adaptation. At the same time, according to the mediation test method proposed by Hayes, the bootstrap method was used to test the mediating effect (Hayes, 2009). Bootstrapping produced an empirical representation of the sampling distribution of indirect effects, in which samples of size *n* obtained were viewed as microcosms of the population and were repeatedly re-sampled during the analysis as a means of simulating the original sampling process (Hayes, 2009). We used a bootstrap procedure involving 3,000 samples and computation of 95% bias-corrected CIs (BCIs) of the estimates. If the path coefficient is within the 95% CI and does not include 0, the direct effect and mediating effect are considered significant.

SPSS (version 22.0; IBM Corporation, The US) and AMOS™ (version 23.0) were used to perform statistical analysis.

## Results

### Descriptive Statistics

Among 1,440 students invited, in total 1,320 valid questionnaires were collected, including 534 male students (40.5%) and 786 female students (59.5%). There were 407 (30.8%) who majored in literature and history, 486 (36.8%) in science and engineering, and 276 (20.9%) in medicine, and the rest of the students 151 (11.5%) in other specialties. The distribution of grades was 331 freshmen (25.1%), 355 sophomores (26.9%), 386 juniors (29.2%), and 248 seniors (18.8%). Regarding sibling status, the number of who being only child was 674 (51.1%), and those being non-only child was 646(48.9%). The scores of the three questionnaires on each demographic variable are shown in Table 1.

**Table 1:** Samples of characteristics (*N*=1,320).

Characteristics		*n* (%)	Total score of parent-child attachment *M(SD)*	Total score of school bonding *M(SD)*	Total score of social adaptation *M(SD)*
Gender	Men	534 (40.5)	73.71(12.68)	36.13(7.32)	154.36(24.86)
	Women	786 (59.5)	75.72(12.36)	36.53(6.84)	151.98(19.04)
Specialty/Field of Study	Literature and history	407 (30.8)	75.04(12.98)	36.72(7.10)	153.49(21.59)
	Science and engineering	486 (36.8)	73.45(12.88)	35.93(7.23)	154.08(25.27)
	Medicine	276 (20.9)	76.81(11.06)	37.24(6.63)	149.91(14.85)
	Other specialties	151 (11.5)	75.73(12.19)	35.19(6.78)	153.34(18.75)
Grade	Freshmen	331 (25.1)	76.02(12.95)	37.38(7.00)	153.23(20.59)
	Sophomores	355 (26.9)	73.64(12.25)	35.65(7.14)	153.92(22.29)
	Juniors	386 (29.2)	74.97(12.86)	35.78(7.01)	150.93(21.98)
	Seniors	248 (18.8)	75.13(11.70)	36.96(7.04)	154.28(21.25)
Sibling Status	One-child families	674 (51.1)	76.16(12.72)	36.93(7.35)	152.38(22.82)
	Non-only child families	646 (48.9)	73.60(12.19)	35.77(6.66)	153.53(20.67)

### The Difference in Parent-Child Attachment Between Genders and Sibling Status

The overall level of parent-child attachment in females (*M*=75.72, *SD*=12.36) was significantly higher than that of males (*M*=73.71, *SD*=12.68; *F*=8.22, *p*<0.01). Difference was also found between sibling status (*F*=13.90, *p*<0.001), and the only-child (*M*=76.16, *SD*=12.72) scored significantly higher than their counterparts (non-only children, *M*=73.60, *SD*=12.19).

In terms of three dimensions (trust, communication, and alienation) of parent-child attachment (See Table 2), females scored significantly higher than males on both parent-child trust and mother-child communication while significantly lower than men on parent-child alienation. Similarly, compared to their counterparts, the only-child had a significantly higher score on trust and communication but significantly lower on alienation.

**Table 2:** Descriptive statistics and *t*-tests for dimensions of parent-child attachment between genders and sibling status (*M±SD*).

		Mother-Child Trust	Father-Child Trust	Parent-Child Trust	Mother-Child Communication	Father-Child Communication	Parent-Child Communication	Mother-Child Alienation	Father-Child Alienation	Parent-Child Alienation
Gender	Male	11.13±2.46	10.91±2.61	22.05±4.74	10.58±2.45	10.46±2.78	21.04±4.86	8.62±3.59	8.75±3.83	17.38±6.97
	Female	11.41±2.31	11.24±2.37	22.65±4.21	10.94±2.51	10.37±2.73	21.31±4.65	8.01±3.37	8.23±3.52	16.24±6.31
	*t(df)*	−2.07(1318)^*^	−2.36(1318)^*^	−2.42(1318)^*^	−2.59(1318)^*^	0.56(1318)	−1.03(1318)	3.15(1318)^**^	2.54(1318)^*^	3.07(1318)^*^
Sibling Status	Only child	11.49±2.43	11.28±2.52	22.77±4.57	11.06±2.49	10.61±2.80	21.67±4.83	8.08±3.50	8.21±3.67	16.29±6.78
	Non-only child	11.10±2.30	10.93±2.42	22.03±4.26	10.51±2.46	10.19±2.69	20.70±4.62	8.45±3.44	8.69±3.64	17.14±6.51
	*t(df)*	3.02(1318)^**^	2.52(1318)^*^	3.02(1318)^**^	4.06(1318)^***^	2.77(1318)^**^	3.73(1318)^***^	1.93(1318)	2.39(1318)^*^	2.34(1318)^*^

SDs are reported in parentheses.

* *p<0.05*;

** *p<0.01 and*

*** *p<0.001*.

Further analyses on subscale scores (mother or father) of each parent-child attachment dimension showed that females scored significantly higher on mother-child trust, father-child trust, and mother-child communication (*p*<0.05) but lower on mother-child alienation and father-child alienation when compared to males. There was no statistical difference between males and females in terms of father-child communication. Regarding sibling status, those only-children scored significantly higher on mother-child trust, father-child trust, mother-child communication, and father-child communication but significantly lower on father-child alienation than those non-only children (*p*<0.05). Regarding mother-child alienation, there was no statistical difference between those only-children and non-only children.

### Correlations Between Parent–Child Attachment, School Bonding, and Social Adaptation

The total score of parent-child attachment was positively correlated with the total score of social adaptation (*r*=0.12, *p*<0.01), and was also significantly positively correlated with the dimensions of social adaptation (interpersonal adaptation, behavioral adaptation, and environmental adaptation; *r*=0.19, *p*<0.01; *r*=0.07, *p*<0.05; *r*=0.19, *p*<0.01), but not with the dimension of self-adaptation. The total score of parent-child attachment was positively correlated with the total score of school bonding (*r*=0.54, *p*<0.01), and was also significantly positively correlated with the dimensions of school bonding (school belonging, teachers’ support, and classmates’ support; *r*=0.51, *p*<0.01; *r*=0.44, *p*<0.01; *r*=0.51, *p*<0.01), the total school bonding score is significantly positively correlated with the social adaptation score (*r*=0.36, *p*<0.01), and it is also significantly positively correlated with various dimensions of social adaptation (self-adaptation, interpersonal adaptation, behavioral adaptation, and environmental adaptation; *r*=0.20, *p*<0.01; *r*=0.33, *p*<0.01; *r*=0.30, *p*<0.01; *r*=0.39, *p*<0.01; See Table 3).

**Table 3:** Correlations between parent-child attachment and the dimensions of social adaptation and school bonding (*N*=1,320).

Variable	1	2	3	4	5	6	7	8	9	10
1. Total score of parent–child attachment	-									
2. Total score of social adaptation	0.12^**^	-								
3. Self-adaptation	0.03	0.90^**^	-							
4. Interpersonal adaptation	0.19^**^	0.73^**^	0.60^**^	-						
5. Behavioral adaptation	0.07^*^	0.80^**^	0.62^**^	0.38^**^	-					
6. Environmental adaptation	0.19^**^	0.85^**^	0.67^**^	0.53^**^	0.60^**^	-				
7. Total score of school bonding	0.54^**^	0.36^**^	0.20^**^	0.33^**^	0.30^**^	0.39^**^	-			
8. School belonging	0.51^**^	0.37^**^	0.22^**^	0.35^**^	0.31^**^	0.40^**^	0.93^**^	-		
9. teachers’ support	0.44^**^	0.41^**^	0.28^**^	0.31^**^	0.38^**^	0.40^**^	0.90^**^	0.80^**^	-	
10. classmates’ support	0.51^**^	0.20^**^	0.06^*^	0.24^**^	0.15^**^	0.25^**^	0.89^**^	0.73^**^	0.65^**^	-

* *p<0.05*;

** *p<0.01*.

### Mediating Effect of School Bonding Between Parent-Child Attachment and Social Adaptation

**Figure 2:**
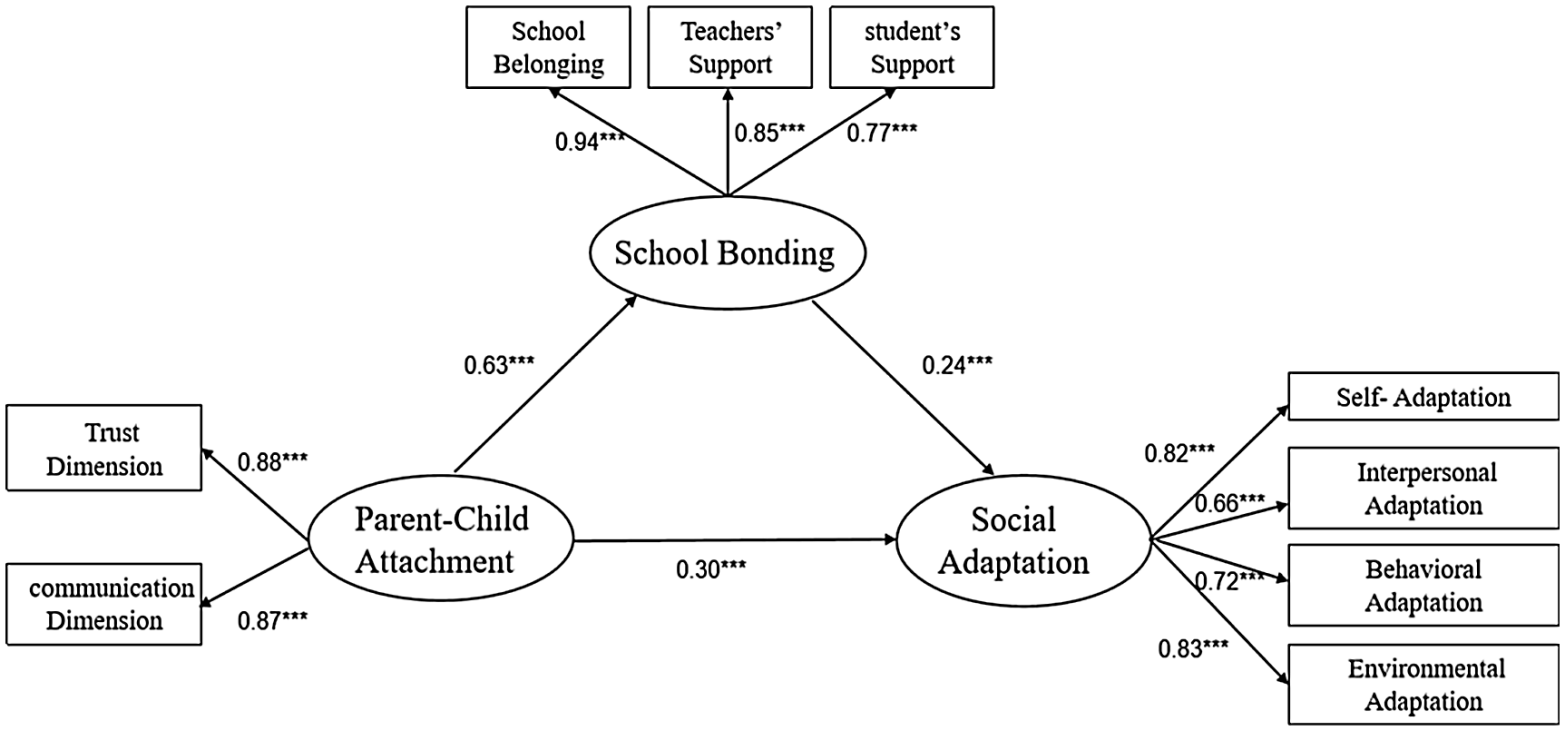
Mediation model of school bonding between parent-child attachment and social adaptation.

The measurement model consisted of three latent factors (parent-child attachment, school bonding, and social adaptation) and 10 observed indicators. The measured variables were the three indicators (three dimensions) of parent-child attachment, i.e., trust, communication, and alienation, and the three indicators (three dimensions) of school bonding, i.e., school belonging, teacher support, and student support and four indicators (four dimensions) of social adaptation, i.e., self-adaptation, interpersonal adaptation, behavioral adaptation, and environmental adaptation. The factor loadings of parent-child alienation were found to be less than 0.5, therefore, the dimension was deleted (Miao et al., 2018). The mediation model suited adequately (Figure 2), with *χ*^2^(112)=391.46, *p*<0.001, CFI=0.95, TLI=0.92, GFI=0.94, NFI=0.94, RFI=0.92, RMSEA=0.08. The bootstrapping showed that the 95% credible intervals of the total effect, direct effect, and indirect effect did not include 0 (see Table 4), indicating that school bonding played a mediating role between parent attachment and social adaptation, with the mediating effect value of 0.15.

**Table 4:** The mediating effect of school bonding on parent-child attachment and social adaptation.

Hypothesis path	*β* (standardized indirect effect)	*SE*	*p*	95% BC bootstrap CI	
				LB	LP
Total effect	0.45	0.36	0.001	0.38	0.52
Direct effect PCA→SA	0.30	0.47	0.001	0.21	0.39
Indirect effect PCA→SB→SA	0.63*0.24=0.15	0.03	0.001	0.09	0.22

*PCA, parent-child attachment; SA, social adaptation; and SB, school bonding*.

## Discussion

Our results showed significant differences in parent-child attachment between genders and sibling statuses among college students. In terms of gender, the overall parent-child attachment of female students was significantly higher than that of male students. Females scored higher than males on mother-child trust, father-child trust, and mother-child communication, while males scored higher on mother-child alienation and father-child alienation. In terms of sibling status (only child vs. non-only child), a higher level of overall parent-child attachment was found among the only child. The communication and trust dimensions of parent-child attachment were found higher among only-child, while alienation was higher in non-only child.

Gender differences in parent-child attachment may be caused by gender characteristics and gender roles of the female, as noted by Raja et al. (1992). Gender roles refer to the position occupied by each gender in the society and group to which it belongs and the specific behavior pattern prescribed and desired by the society and group (Lindsey, 2016). In Chinese culture, people believe that boys should be responsible, independent, and strong. This kind of culture educates boys to be independent and emotion-controlled, leading to less communication with their parents. This gender-specific characteristic will become manifest as they age. For girls, they are expected to show more “femininity,” including being more emotional, gentle, caring for others, and therefore parents’ expectation of their achievement will be lower than that of boys (Ho, 1981; Buist et al., 2002; Song et al., 2009). Such culture facilitates more intimate behaviors with their parents, and thus girls develop more trust in their parents, communicate better with their parents, and have a lower sense of alienation (Laible et al., 2004; Song et al., 2009). Thus, culture-related parents’ expectations for their children may lead to gender-specific parenting styles and parent-child interaction, which in turn contributes to the gender differences in the parent-child attachment (Guo et al., 2018). According to Resource Dilution Model, the development of each child is dependent on family resources and their allocation. As the number of children in a family increases, the resources provided by parents to each individual will be “diluted” (Blake, 1981; Downey, 2001). Therefore, the difference in parent-child attachment between only-children and non-only children can be clarified by the fact that parents in non-only child families need to take care of more than one child, parents’ care and protection are distributed unevenly across siblings, and that this distribution is determined by the gender and number of siblings, which make them easily distracted and unable to meet the needs of each child in a timely manner (Kitamura et al., 1998; Wang, 2005; Foster et al., 2020). The finding that parent-child communication interactions, as well as emotional exchanges, decrease as the number of children increases is consistent with other retrospective studies (Kitamura et al., 1998; Someya et al., 2000; Guo and Luo, 2019). Compared to the only child, non-only children have lower levels of communication and trust with their parents and a higher level of alienation (Zhao et al., 2015).

Our correlation analysis showed that parent-child attachment was positively correlated with social adaptation and its dimensions among college students, which is consistent with the results of Eberly and Montemayor (1998) and Mikulincer and Shaver (2015). This suggested that social adaptation can be improved by promoting parent-child attachment. Nickerson and others pointed out that a high level of parent-child attachment is a protective factor for the physical and mental health of college students, and good parent-child attachment is conducive to the development of social adaptability to reduce anti-social behaviors (Nickerson and Nagle, 2004). Similarly, Kenny and Hart (1992) also found that high-quality parent-child attachment can help individuals obtain more supportive resources from others or the external environment, and also help individuals identify their effective resources and enhance their ability to adapt to the environment. In addition, a study conducted by Oldfield et al. (2016) showed that individuals with secure attachment incline to form positive social adaptation and reduce the intensity and frequency of negative adaptation. Hence, the quality of parent-child attachment will affect college students’ perception of social support in the familiar environment (family, friends, and teachers), and this perception will further enrich and activate college students’ psychological resources and promote their positive social adaptation (Crespo et al., 2013).

The mediation analysis showed that school bonding plays a partial mediating role between parent-child attachment and social adaptation, which implied that parent-child attachment cannot only directly affect the level of social adaptation of college students but also indirectly affect social adaptation through school bonding. The internal working model of parent-child attachment is formed in the process of parent-child interaction (Bowlby, 1988). Such interaction helps construct one’s cognition about self, others, and the environment, and such experience will enhance the cognition-emotion link that subsequently improves interpersonal relationships with others (Bradford and Lyddon, 1993; Thompson and Raikes, 2003). Precisely speaking, a good parent-child attachment means that there is effective communication between parents and children, high-quality care received, positive self-other cognitive schema generated, and belief in love and trust (Rostad and Whitaker, 2016; Mikulincer and Shaver, 2017). College students with good parent-child attachment are more likely to feel the care and acceptance from peers, teachers, and school administrators in school, which is conducive to establishing positive emotional connectedness with teachers, high-quality friendship with peers, and then a sense of belonging towards the school (Kerpelman et al., 2013; Ye et al., 2020). All these contribute to the development of social adaptability. Therefore, the parent-child attachment could impact college students’ social adaptation through school bonding.

A sense of belonging is the feeling of positive relationships with others and is the fundamental human need (Walton and Brady, 2017). Consistent with earlier studies by Mikulincer, Nickerson, and Gorrese et al., which also found that high parent-child attachment is beneficial to the development of a positive mode of interaction with others, gain the ability and psychological resources to pay more attention to the needs of others, show stronger interpersonal skills, obtain the attention and acceptance of peers and teachers to promote the formation of the support from the classmates and teachers that is an important part of school bonding (Nickerson and Nagle, 2004; Gorrese and Ruggieri, 2012; Mikulincer and Shaver, 2015). A study of Eisenberg et al. (2003) showed that students with a high level of school bonding tend to present higher academic achievement and lower dropout rate, and they suggested a sense of belonging towards school plays a more prominent role in social adaptability development. School isolation and loneliness not only impair subjective well-being but also intellectual achievement and immune function, and psychosomatic health (Walton and Cohen, 2007). Students with a strong sense of belonging to their school will interact more with their peers and teachers on campus, forming better relationships, facilitating their school integration, and further contributing to their well-being, performance, and social adaptation (Wilson, 2006). Walton and Cohen (2011) also found that a sense of belonging is a psychological advantage, which can significantly promote the academic performance, self-reported health, and well-being of college students. This is because college students with a strong sense of belonging have a willingness to abide by school rules and regulations, and are also more inclined to develop close friendships with classmates and actively participate in school activities, which in turn may help reduce aggressive behavior, gain a greater sense of belonging and opportunities for growth, and thus enhance social adaptation (Broda et al., 2018).

## Strengths and Limitations

Strengths of this study encompass the use of validated scales to measure studied variables, and of mediation analysis that aids in understanding the potential pathway of school bonding between parent-child attachment and social adaptability. There were also several limitations. Firstly, although our cluster sample was drawn from four universities, the generalizability of the results needs to be further verified. Secondly, social adaptation is a process of constant development and change, and the cross-sectional study adopted in this study cannot well reflect its dynamics. Longitudinal studies can be considered for further research in the future. At the same time, the self-report method adopted in this study cannot avoid the influence of social desirability. In the later stage, behavioral observation and experimental methods can be used to further verify the relationship between variables.

## Conclusion

In summary, both parents and schools play a crucial role in helping college students develop higher social adaptation. Corresponding measures aimed to promote college students’ social adaptation should be considered at both family and school levels. It is important that parents should provide strong emotional support for their children by adopting the parenting style that can foster parent-child trust, enhance communication between parents and children, and minimize alienation to establish parent-child attachment. It is crucial to strengthen the parent-child attachment bond in non-one-child families. The increase of siblings can weaken the specific influence of parent-child communication and interaction on each child. For non-only-child families, parents should pay as much attention to each child as possible, give them equal care, engage in emotional interaction with their children, establish an intimate relationship to avoid the adverse impact of “diluted” resources so as to improve the quality of parent-child attachment. In the meantime, teachers should actively empathize with students, understand their psychological characteristics, respect them, stimulate their creativity, engage in open communication about life issues and problems of young people, treat them with care and respect, and acknowledge their needs and opinions. It is also important to take the initiative to establish friendly teacher-student relations for students, and to build up platforms facilitating student participation in school activities. For example, counselors and class leaders should play a role in organizing meaningful class activities and strengthening campus culture this may help students gain a sense of school belonging which can potentially reduce problem behaviors, and then enhance adaptability. As for college students, they may also need to consciously raise the awareness of themselves and their emotion and behavior, as well as actively engage in the interaction and communication with parents, teachers and peers in order to enhance social adaptation.

## Data Availability Statement

The authors acknowledge that the data presented in this study must be deposited and made publicly available in an acceptable repository, prior to publication. Frontiers cannot accept a manuscript that does not adhere to our open data policies.

## Ethics Statement

The studies involving human participants were reviewed and approved by Ethics Committee of Kangda College of Nanjing Medical University. The patients/participants provided their written informed consent to participate in this study. Written informed consent was obtained from the individual(s) for the publication of any potentially identifiable images or data included in this article.

## Author Contributions

HY conceived the original idea and designed the whole research process. HY, W-KM, and SQ collected and cleaned the data. HY, W-KM, SQ, FH, and HZ did the data analysis and data interpretation. HY, W-KM, SQ, and FH wrote the first version of the manuscript. HY and W-KM contributed to the administration of the project. W-KM, CJPZ, and FH critically reviewed, edited, and revised the manuscript for important intellectual content. All authors contributed to the interpretation of the results and the final manuscript and discussed and agreed on the implications of the study findings and approved the final version to be published. All authors contributed to the article and approved the submitted version.

## Funding

This work was supported by Nanjing Medical University Kangda College 2019 Scientific Research and Development Fund Key Project (KD2019KYJJZD004) , Nanjing Medical University 2019 Annual Education Research Project (2019ZC015), and Jiangsu Education Bureau’s 2017 College Philosophy and Social Science Fund Project (2017SJBFDY515). This study was also funded by the Research Talent Training Program of Kangda College of Nanjing Medical University.

## Conflict of Interest

The authors declare that the research was conducted in the absence of any commercial or financial relationships that could be construed as a potential conflict of interest.

## Publisher’s Note

All claims expressed in this article are solely those of the authors and do not necessarily represent those of their affiliated organizations, or those of the publisher, the editors and the reviewers. Any product that may be evaluated in this article, or claim that may be made by its manufacturer, is not guaranteed or endorsed by the publisher.
